# Acceptability and feasibility of the mHealth intervention ‘MyDayPlan’ to increase physical activity in a general adult population

**DOI:** 10.1186/s12889-020-09148-9

**Published:** 2020-06-29

**Authors:** L. Degroote, D. Van Dyck, I. De Bourdeaudhuij, A. De Paepe, G. Crombez

**Affiliations:** 1 Department of Movement and Sport Sciences, Ghent University, Ghent, Belgium; 2 Research Foundation Flanders, Brussels, Belgium; 3 Department of Clinical-Experimental and Health Psychology, Ghent University, Ghent, Belgium

**Keywords:** eHealth, mHealth, Self-regulation, Interview, Physical activity, Feasibility, Acceptability

## Abstract

**Background:**

Electronic health (eHealth) and mobile health (mHealth) interventions have the potential to tackle the worldwide problem of physical inactivity. However, they often suffer from large attrition rates. Consequently, feasibility and acceptability of interventions have become important matters in the creation of e- and mHealth interventions. The aim of this study was to evaluate participants’ opinions regarding acceptability and feasibility of a self-regulation, app-based intervention called ‘MyDayPlan’. ‘MyDayPlan’ provides an innovative daily cycle providing several self-regulation techniques throughout the day that guide users towards an active lifestyle via various self-regulation techniques.

**Methods:**

Semi-structured interviews were conducted with 20 adults after using the app for 2 weeks. A directed content analysis was performed using NVivo Software.

**Results:**

‘MyDayPlan’ was well-received and seems to be feasible and acceptable with inactive adults. The straightforward lay out and ease of use of the app were appreciated. Furthermore, the incorporation of the techniques ‘action planning’, and ‘prompting review of behavioral goals’ was positively evaluated. However, the users gave some recommendations: implementation of activity trackers to self-monitor physical activity could be of added value. Furthermore, increasing intuitiveness by minimizing text input and providing more preprogrammed options could further increase the ease of use. Finally, users indicated that they would benefit from more guidance during the “coping planning” component (barrier identification/problem solving), for example by receiving more tailored examples.

**Conclusions:**

Based on these findings, adaptations will be made to the ‘MyDayPlan’ app before evaluating its effectiveness. Furthermore, involving potential end users and evaluating acceptability and feasibility during the development of an e- and mHealth intervention is key. Also, creating interventions with a large ease of use and straightforward layout that provides tailored support during action and coping planning is key.

## Background

Regularly engaging in physical activity (PA) is important to prevent the development of non-communicable diseases (NCDs), amongst which heart disease, stroke [1], type II diabetes and breast and colon cancer [2]. Despite these health benefits, many adults do not meet the health recommendations for PA. The World Health Organization states that adults between 18 and 65 years should at least do 150 min of moderate-intensity PA, or at least 75 min of vigorous-intensity PA throughout the week, or an equivalent combination of both [3]. In 2018, Guthold et al. stated in The Lancet Global Health that more than one in four adults worldwide (28% or 1.4 billion people) are physically inactive [4]. Consequently, lifestyle interventions that promote PA in a large number of people at low costs are required. E- and mHealth interventions may be a promising avenue to achieve this.

The concept of eHealth is based on the use of information and communication technologies in support of health and health-related fields including healthcare, health surveillance and health education, knowledge and research [5]. The concept of mHealth is a sub-segment of eHealth, and refers to the use of mobile communication devices (e.g. mobile phones, tablet computers and personal digital assistants (PDAs)), and wearable devices (e.g. smart watches) [6]. Interventions using e- and mHealth have potential to change health behaviours, such as PA, dietary habits and smoking [7–9]. Because of the widespread use of the internet and related technologies (e.g. smartphones, smartwatches, tablets,..) eHealth interventions can reach a large number of people in a cost-effective way [10, 11]. Notwithstanding, studies often report high attrition rates [12–15] and small intervention effects [16]. These problems need to be addressed in order to employ the full potential of e- and mHealth interventions. First, high attrition is likely due to the intervention not being matched to the needs, goals and expectations of users. This may be avoided by involving potential users during the entire cycle of intervention development [17, 18]. Second, research revealed that theory-based interventions are more effective at changing health behaviours than atheoretical interventions [19–21]. So, interventions should be grounded within and informed by theoretical models [22]. Theory-based interventions also provide a useful framework for targeting and investigating the underlying processes of change. Behaviour effects may be increased by targeting several processes, for example, described in the Health Action Process Approach (HAPA) [23, 24]. HAPA is a process model that identifies several determinants contributing to health behaviour change. More specific, HAPA categorizes these determinants into two phases, a motivational (pre-intentional) phase contributing to forming an intention towards behaviour change and a volitional (post-intentional) phase contributing to the translation of an intention into actual behaviour change.

Self-regulation techniques may be used to target behaviour change processes as described in the HAPA model. Two earlier HAPA based e- and mHealth interventions, called ‘MyPlan 1.0’ and ‘MyPlan 2.0’, adopted several self-regulation techniques to guide users towards more PA, less sedentary behaviour and more fruit and vegetable consumption [25–28]. ‘MyPlan 1.0’ implemented these self-regulation techniques in a website, whereas ‘MyPlan 2.0’ used both a website and a mobile application. In ‘MyPlan 1.0’. and ‘MyPlan 2.0’. pre-intentional processes were addressed by providing personal feedback to raise awareness and motivating participants to change their level of PA, sedentary behaviour and fruit and vegetable consumption. Post-intentional processes that bridge the gap between intentions and behaviour were addressed by inviting participants to make an action plan, by offering the possibility to identify difficult situations, hindering factors and the relevant solutions to overcome these (i.e. coping planning). Participants were finally advised on how to self-monitor their behaviour (e.g. using an agenda) and to pursue their health goals as stated in their action plan [29]. Using a quasi-experimental trial, ‘MyPlan 1.0’ has proven to be effective in improving PA and fruit and vegetable intake in the general population in a general practitioner (GP) setting [25, 26]. Effectiveness of the programme has also been demonstrated in recently retired Belgian adults by conducting a randomized controlled trial (RCT) [29]. However, high attrition rates were observed in both studies and researchers experienced problems with the implementation in the setting of general practitioners [25, 26, 29]. The programme was therefore further developed into ‘MyPlan 2.0’ for use as a stand-alone intervention. In order to reduce attrition, end-users were involved by conducting semi-structured interviews, think aloud sessions and questionnaires [30–33]. Based on these, several adaptations were made, leading to the development of ‘MyPlan 2.0’: the intervention was shortened, the text was limited, information sheets were substituted by a quiz, and the layout was changed. Furthermore, rationales were provided for the implementation of different self-regulation techniques, specific instructions were given during action planning and barrier identification/problem solving, and general tips and tricks were provided. Moreover, success stories of other users were added. Furthermore, a mobile application was added, as a supplement to the website. Three RCTs revealed that ‘MyPlan 2.0’ is effective in older adults [34], a 50-plus population and type 2 diabetes population [27]. It is currently being tested in a general adult population [35].

‘MyPlan 1.0’ and ‘MyPlan 2.0’ both use a time window of 1 week for action and coping plans. Such timescale may be too long to take into account the often (un)foreseen changes from day to day. Qualitative research with ‘MyPlan 1.0’ and ‘MyPlan 2.0’ confirmed this potential problem. Users reported that the formulation of actions and coping plans for the upcoming week was difficult because they were not able to anticipate contextual factors that fluctuate from day to day (e.g. location, weather, agenda, mood, health status, motivation,…). Consequently, several action and coping plans lacked specificity and instrumentality [36]. Instrumentality refers to the degree that a plan is goal-directed [37]. Specificity refers to the degree that a plan includes a high amount of details about what, where, when, how and with whom the plan will be performed [38]. Highly instrumental and specific action and coping plans increase the likelihood that individuals perform an intended behaviour. This notion is based on the idea that individuals who describe the anticipated behaviour and context with sufficient precision will recognize the critical situation more easily and will therefore be more likely to respond as they had previously planned [38]. In order to ensure specific and instrumental action and coping plans, individuals must have sufficient insight into the upcoming context in which they want to be physically active. Therefore, action and coping plans for a short timescale (daily) may better match the reality of contexts varying from day to day. Furthermore, short (1-day) cycles of action and coping planning allow individuals to ‘learn by doing’, meaning that they gradually tailor their action and coping plans based on their experience. Hence we developed the mHealth intervention ‘MyDayPlan’. The intervention is based on the HAPA model (in particular the post-intentional phase of the HAPA model). ‘MyDayPlan’ offers users a range of self-regulation techniques influencing several determinants presented in the HAPA model and guiding users to behaviour change. Users go through a cycle of self-regulation techniques in 1 day. We presume that a one-day cycle allows people to set more achievable, realistic action and coping plans by taking into account the anticipated opportunities/obstacles of the day.

The aim of this study was to gain insight into users’ thoughts and impressions on ‘MyDayPlan’ using semi-structured interview after having used the app in their everyday lives for 2 weeks. Allowing to evaluate feasibility and acceptability of the intervention before evaluating its effect on users’ level of PA in further research.

## Methods

### Participants

Healthy participants between the age of 18 and 65 years were recruited using purposive sampling. More specifically, participants of previous studies who indicated to have interest in other research were contacted by email. Inclusion criteria were age (18–65 years old), having no current physical limitations, medical conditions, or psychiatric conditions, owning a smartphone using Android as operating system, having internet access, having the ability to understand and speak Dutch. Exclusion criteria included achieving the PA guidelines of 150 min moderate-to-vigorous physical activity (MVPA) per week. This was measured by the International Physical Activity Questionnaire-Long Form (IPAQ-LF, 7d version). The IPAQ-LF asks participants to report the frequency and duration of activities in the last 7 days. Earlier research indicated that IPAQ is a reasonably reliable valid measurement tool for measuring habitual PA [39, 40].

In total, 47 people showed interest in the study, and were invited via e-mail to fill out the IPAQ. Only those who did not meet the guideline of 150 min MVPA per week were selected to participate [25, 41]. This was the case for 22 participants. All participants provided a written informed consent. The study protocol was approved by the Ethics Committee of the University hospital of Ghent (study number: B670201835315).

### Description of ‘MyDayPlan’

‘MyDayPlan’ is an mHealth intervention, consisting of a mobile application, targeting PA in adults from the general population. The intervention is based on ‘MyPlan 1.0’ and ‘MyPlan 2.0’ [25, 27, 28].

Screenshots of the application can be found in Additional file 1. The one-day cycle of ‘MyDayPlan’ is as follows: each morning at 8 am, participants receive a notification to go to the app as soon as they start their day. First, they are asked to formulate an action plan, consisting of specific actions that they want to do that day to be more physically active. They are asked to formulate actions that they could do on top of the activities they routinely do. They have the possibility to formulate specific action plans within a maximum of 2 out of 4 domains (transport, household, work/school, leisure time). This maximum was chosen to limit participant’s burden and to ensure fast sessions with the app. For each domain participants can formulate one or more actions. After formulating specific actions, participants indicate how difficult they think it would be to perform the predetermined action plan. After this, users are asked to formulate possible barriers and possible solutions to overcome these. If they need some inspiration, some examples are provided. After the coping planning, the morning session is finished. For the evening session, all users receive a notification at 8 pm. They are again asked to go to the app at the end of the day, and to reflect on their physical activities for that day. More specifically they are asked to indicate to what extent they executed their action plans. If they executed their action plans, they are asked to think about the factors that have helped and to consider these for the next day. If they did not reach their goal, users are asked to think about the factors that hindered the execution of their action plan. They were encouraged to take these into account for the next day. Table 1 gives an overview of the behaviour change techniques included in ‘MyDayPlan’.

**Table 1:** Overview of the self-regulation techniques implemented in the ‘MyDayPlan’ app

Self-regulation Technique	Implementation mode
Action planning	Participants are asked to formulate specific actions each morning to be more physically active during that day.
Barrier identification/problem solving (=coping planning)	Participants are asked to formulate specific barriers and possible solutions to overcome these barriers.
Reviewing goal achievement	Participants are asked to reflect on whether they executed their action plan. They are also asked to reflect on what helped to execute their action plan or on what hindered them to execute their action plan.

### Study design

**Fig. 1:**
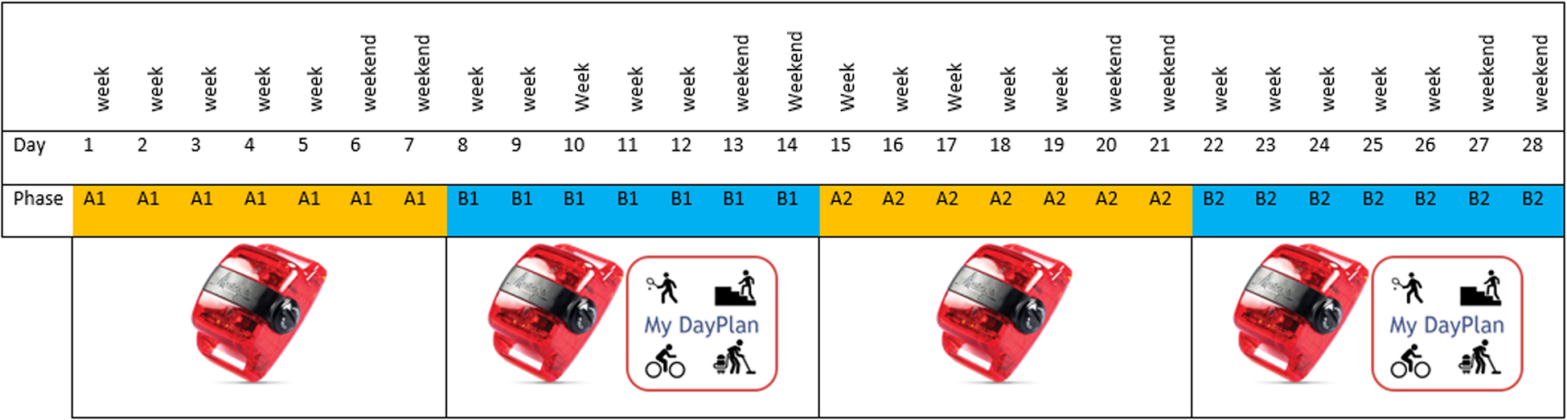
Specific ABAB reversal design

In this study, we used an ABAB reversal design. As we plan to test the effectiveness of ‘MyDayPlan’ in the future by using this design, this study was used to pilot test this design. However, results on feasibility of the design and effectiveness of ‘MyDayPlan’ were not included in this paper. The ABAB reversal design is a single case design investigating the effect of the intervention by alternating the presentation (B) and removal (A) of the intervention during a period of consecutive days. The study flow is depicted in Fig. 1. The study duration was 4 weeks for all participants. The duration of each of the 4 different phases of the design was 1 week (7 days). During the first (A1 = baseline phase) and the third phase (A2 = reversal phase), participants were instructed not to use the ‘MyDayPlan’ app. During the second (B1 = intervention phase) and the fourth (B2 = intervention phase) phase, participants were instructed to use the ‘MyDayPlan’ app. During the entire study, participants wore an ActiGraph accelerometer to measure their daily level of PA. However, in this study, the effectiveness of ‘MyDayPlan’ was not yet examined, so the PA measurements were not considered. Although the participants completed the four phases during this study, only during two phases (B1 and B2) the ‘MyDayPlan’ app was used. Consequently, the semi-structured interview afterwards assessing feasibility and acceptability of the app only covered these two intervention weeks during which the app was used.

Prior to the start of the study (during the week before the study) participants were visited at home. During this first home visit, participants were informed about the study and provided with an ActiGraph accelerometer and a charging cable. Also, the ‘MyDayPlan’ app was installed on their mobile phone and phone settings were checked to allow notifications from the app. To avoid bias during the A1 phase (=baseline) no substantive information about the app was given during the home visit. A day before the start of the study, participants were given a login for the ‘MyDayPlan’ app through email. They were asked to log into the app in the morning of the first day of the study. From that moment on, the ‘MyDayPlan’ app provided all communication with the participant. Each morning, participants received a notification with the message to either use the app that day, or not use the app that day. After completing the 4 weeks of study, a second home visit was scheduled to collect the ActiGraph and the charging cable. During this second home visit, a semi-structured interview was conducted focusing on acceptability and feasibility of the ‘MyDayPlan’ app.

### Semi structured interview

The interviews were audio recorded with permission of the participants. The questions and content of the semi-structured interview were based on previous research [42]. The main topics addressed during this interview were: design of the app (user-friendliness, layout and time efficiency); usefulness of the app (i.e. personal relevance, awareness and behaviour change elicited by the app); perception of the behaviour change techniques used in the app (action planning, coping planning and monitoring) and overall recommendations of the users. The interview guide can be found in Additional file 2. The average duration of an interview was 20 min (range: 12 min–27 min).

### Data analysis

Interviews were transcribed verbatim (https://transcribe.wreally.com), and a directed content analysis was performed using NVivo Software (QSRInternational, Melbourne, Australia, Version 11, 2015) [34]. The coding scheme of the directed content analysis was based on previous research with ‘MyPlan 1.0’ and ‘MyPlan 2.0’ [42].

When a fragment of the interview did not fit any of the predefined categories, a new category was created. Themes that did not contain enough data, meaning only 1 fragment, were not withheld. This resulted in the coding scheme that is presented in Additional file 3. Coding was performed independently by two researchers (LD and SW).

## Findings

### Demographic characteristics

Of the 22 participants, 20 completed the study. Two participants experienced technical problems during the use of the ‘MyDayPlan’ app. More specifically, the app froze on their smartphone and was no longer accessible. This problem could not be solved. No semi-structured interview was conducted with these participants. Characteristics (gender, age and MVPA) of the remaining 20 participants are presented in Table 2.

**Table 2:** Participants’ characteristics

Characteristics	Participants (*N* = 20)
**Gender, n (%)**	
Men	7 (35)
Women	13 (65)
**Age in years, mean (SD), range**	39 (17.6), 18–64
**MVPA in min per day, mean (SD), range**	13.2 (9.6), 0–23.4

### Interviews

All the information described below was obtained during the semi-structured interviews conducted after the testing period.

### Design of the app

#### User friendliness

Most of the users (14/20) perceived the app as user friendly and easy to use. Users highlighted the fact that it was intuitive and straightforward with a good flow.

*It is an easy app to use. It was all very clear what was expected from me. [Woman, 54 years]*

*In itself it was certainly not a difficult app. I was a bit scared, because it included a manual, but eventually I even didn’t use it. [Woman, 33 years]*

However some users (5/20) indicated to have experienced practical difficulties when formulating specific actions and barriers. These difficulties lowered the app’s intuitiveness.

*If you go to another day and you want to formulate barriers, it will still be there from the previous day. You then have to delete it. [Man, 38 years]*

*I had a lot of trouble working with it in the beginning: after formulating actions you automatically came across the barriers screen. When I was inattentive here, I was automatically too far without setting up the barriers and I could not go back to the barriers screen. I had not formulated any barriers for those days. The first days I have struggled with this. [Woman, 29 years]*

*What I did find annoying was that at some point you can't adapt the text you put into the app. When you have entered something and you press OK, you can't go back to adjusting which is quite annoying in certain cases. [Man, 36 years]*

In addition to this problem, two other technical problems were mentioned by a few participants. A first problem concerned the notification (3/20), and a second problem was related to the graph depicting the goal achievement of the past days (1/20).

*The notification! The notification has been a problem, especially the second week I had to use the app. The notification just didn't get through. The problem is that you are in a hurry in the morning and you just do not think about the app. You go to work and during the day you realize: Oh, I forgot to fill out the morning session of the app. Therefore, a notification is really useful. [woman, 54 years]*

*The graph wasn't right. When I wanted to look back on the past few days, it didn't match with what I had actually done. During the second week the graph of the first week remained. So that was of no use to me at all. [Man, 29 years]*

#### Lay-out

Although the lay-out of the app is not the main concern during the development of the ‘MyDayPlan’ app, sufficient attention should be paid to how users experience it. An attractive layout can contribute to reducing attrition during the use of the app. All users perceived the layout of the app as basic and simple. This was experienced as positive by most users (14/20).

*Simple, I thought the layout was good and clear. For me personally it shouldn't be with a lot of frills. In that respect the app certainly meets my expectations. [Woman, 28 years]*

*It's simple and easy. It's just a functional app. It preserves its function. That's what it's for and that's what you use it for. It's clear what you have to do. [Man, 22 years]*

However, other users (4/20) indicated that the layout was too simplistic and that they would have liked a more attractive layout.

*For the time being, it's pretty basic. I think that for the youth, young people, even though I can't count myself among the youth anymore, it's not really super hip. I don't think it matters that much to older people, but if you want younger people to work with it, I think it should be more attractive, needs more colour, cooler shapes, cooler fonts, things like that...[Man, 33 years]*

*Not stimulating at all… this seemed very old-fashioned and boring to me. I can of course understand that an app is not easy to make or program, but if you compare it to other apps, the difference in design is really huge.[Man, 25 years]*

#### Time efficiency

Most users (17/20) appreciated the time efficiency of the app, indicating they needed from 1 to 5 min in the morning and the evening to fill out the app. One user indicated spending on average 15 min in the morning and 15 min in the evening on the app. For most users, time was not perceived as a barrier to engage in using the app.

*Time is negligible, you mustn’t leave it at all for that. [Woman, 52 years]*

*I think the time efficiency is good! I lost very little time on it, especially in the evening, it's just a matter of writing it down: that's why I (didn't) succeed today. [Man, 33 years]*

*The amount of time is not much, but sometimes it is… because you get the notification at 8 o'clock, that didn't fit my working schedule. If I start with an early shift, I get up at 5 o'clock in the morning, at 6.30 o'clock I'm already at work… and then I actually have to think about my app during my coffee break… And those things didn't always go so well….[Woman, 54years]*

Some users (3/20) indicated that time efficiency could be better.

*Honestly... it took too much time and it was not used optimally. If this app has to be accessible to people who work and don't have much time in the morning, it won't work for me. I have time in the morning to quickly fill in some multiple-choice questions, but not to write texts. [Woman, 27 years]*

*No, no, I wouldn't necessarily quit for the time spent. If it could go a bit faster, that would be even better... And it would be even more advanced with voice control. For example, I would have found it easy if you could tell it in the car what you wanted to do while you were on the road. [Man, 33 years]*

### Usefulness of the app

#### Personal relevance

The vast majority (15/20) of users experience the ‘MyDayPlan’ app as personally relevant, an intervention that could be of benefit to them.

*Yeah, because my biggest excuse for not exercising is time. The app makes me aware of the fact that, for example going to work by bike is an option. It might take a bit longer, but you've already done some sport implemented in your usual daily schedule. With a small investment of time, you've already had some exercise. Also because you have the subdivision of: at home, at work, in your free time. That subdivision of domains made me think about the possibilities to exercise more. [Man, 33 years]*

*For me it is relevant to make me aware of what I do. But I am now in a phase in my life where I am also busy with 'how much do I actually move'. That's probably why I've come to you now. And it is an extra tool that can help me with that, like a pedometer actually.[Woman, 54 years]*

Two users indicated that the app is not personally relevant for them. They also indicated what would be more relevant to them.

*I think it should be less non-committal and more challenging for someone like me. Not so much an obligation, but in the sense of: you took 12,000 steps yesterday, try 12,500 today? But also with a scoreboard, for example, or a medal you can earn, more of a game element...? [Man, 25 years]*

*No, based on the questionnaire that had to be filled in beforehand, I would expect that more possible, tailored goals could be offered. If you notice, like me, that the minutes of movement per day are very low, I would expect more low-threshold proposals to gradually have an effect, e.g. standing while working, break up long sitting periods,.. [Woman, 28 years]*

#### Awareness and behaviour change elicited by the app

All users perceived that the app had a positive effect on the awareness of the extent to which they are physically active. Some users (12/20) also indicated that they perceived the app as motivating and stimulating behavioural change.

*Yeah, if you set a daily goal that you think is very achievable but you do not achieve it at the end of the day, you don't think you're doing a good job, do you? Then you are aware of the fact that you have a very inactive life, in my case. [Woman, 52 years]*

*It actually made me aware that I have a very busy life. It's strange to say, but it is true. In your daily life you are stuck in your routine and you have to try to find time. I noticed this during the use of the app. One of the easiest goals I could set is to get my son to school by bike. It means that I have to drive home from work with the car, then taking his bike on my bike to school, then riding back home with him. Then, I still have to pick up my daughter from nursery. To do this I have to leave the bike back at home to leave by car. That's just very time-consuming. [Man, 33 years]*

*Much more aware! For me, what motivated me the most was the ‘big brother effect’. Knowing that you can watch as a researcher. In the future, it may be an option that your friends or family have the possibility to see what you do or don't do to achieve your daily goals. This could be a solution to keep the ‘big brother effect’. I also felt morally obliged to do my best, purely because you (researcher) could see what I did. [Woman, 27 years]*

Most users (14/20) experienced problems putting their intention into action. Notwithstanding, if users indicated that the app had helped them to be more physically active, many users also indicated that they were convinced that this had changed their behaviour only in the short term, but that they had doubts about the effect on their behaviour in the long term.

*At the moment I'm sure I'm moving more! Afterwards, I am not convinced that I will be able to keep this up. [Man, 26 years]*

*The app, I think, has enabled me to move more. What does strike me is that it all takes a lot of effort to move more. So I do wonder if I could keep this up in the long run. I'm a bit afraid that I wouldn't like to fill in the app permanently every day. And without the app I wouldn't be able to motivate myself to think about all this. [Woman, 52 years]*

### Perception of used behaviour change techniques in the app

#### Action planning

Examples of the action plans formulated by the participants are the following.

- *I will place my car a bit further on the parking lot today to do the rest of the distance on foot. (Man, 26 years)*
- *Every time I receive a call, I will stand up and walk around as long as I am on the phone. (Man, 38 years)*
- *This evening I will not place the firewood basket next to the fireplace, but a little further. So I have to take more steps to get wood. (Woman, 36 years)*
- *Today I will bring fresh laundry upstairs several times instead of saving everything and taking it all at once (Woman, 54 years)*

The action planning module was experienced as motivating and useful by many users (12/20). By formulating concrete actions that they wanted to achieve that day, many users managed to increase their efforts. However, formulating concrete actions was perceived to be a difficult task by most users (12/20). Users wrongly assumed that different and creative actions should be set up every day. This assumption meant that a lot of users, who were less creative, experienced a lot of trouble with that. In addition, some users (3/20) mentioned that it was not clear to them how to create a good and achievable action.

*The planning of actions surely helped me to move more. Thinking about what I can do. During the first few days I have tried to formulate something extra. But actually I could have just said, I'm just going to clean the house and that way I'll have enough exercise. It gets harder and harder to come up with things. In the beginning you're going to make an effort to do that and that... but at the end I formulated the same and rather simple actions. [Woman, 28 years]*

*I liked setting those goals and actions in the beginning, but every day... It might be more fun to do that 3 times a week... Looking for something new every day is also difficult. More examples and ideas would be helpful. Because it all has to come from yourself, I found it less useful. I didn't really think it was an added value, because the app doesn't really propose things. [Man, 38 years]*

*I really thought that formulating the actions was an added value and, on top of that, quite simple, but I do think that it is because of my education that we have learned to formulate such goals. I think that it can be more difficult for a layman and that a greater variety of tips can work there. [Woman, 27 years]*

*I perceived the app as fairly intuitive, easy to use. However, the only thing I personally found difficult is that there are very limited amount of examples when formulating a specific action. [Woman, 27 years)*

#### Coping planning

Examples of the coping plans formulated by the participants are the following.

- *Barrier: heavy rainfall*
  *Solution: good raincoat*
- *Barrier: bad weather*
  *Solution: run with music on to make it more fun*
- *Barrier: be too tired*
  *Solution: keep thinking about the beautiful figure it will bring about*
- *Barrier: being too busy working so I forget to go for a run*
  *Solution: put the run in the agenda and also set an alarm*
- *Barrier: being very crowded on the parking lot of the supermarket, so I have to put the car closer to the entrance*
  *Solution: walk through all of the corridors in the supermarket to compensate.*

The opinions about the coping planning module were varied. Most users (13/20) agreed that it was difficult to anticipate on potential barriers that could hinder the achievement of their goal. Some users (5/20) indicated that more guidance throughout this module would be helpful. Some users (9/20) indicated that thinking about barriers and possible solutions in advance was very helpful:

*Yes, I thought that was an added value: the fact that you just think about possible barriers. Despite the fact you cannot always prevent them from occurring, but by thinking about it, you'll be able to avoid a lot of things. [Woman, 29 years]*

However, some of the users (7/20) perceived the coping planning module as not useful and even superfluous. They indicated that thinking about possible barriers and solutions is already contained in the reflection and formulation of concrete actions for that day.

*I think it is unnecessary to think about this, because in the end, you are proposing something that is within your means. For example, if you know that you have visitors that afternoon, you will not formulate 'walking with the dog in the afternoon' as one of the actions for that day. Also the weather for example, in the morning you also know what the weather is going to be like? [Man, 22 years]*

*That module is less fun. Since it's often about things you can think of yourself, without using the app. It would be nicer if possible solutions are provided by the app. [Man, 25 years]*

#### Reviewing goal achievement

Most users (17/20) highly appreciated the ‘reflection’ module and experienced it as stimulating and motivating.

*I think this must be stimulating for everyone. This gives you a clear visual image whether or not you are doing well. And certainly if you had the ability to see your results, compared to the average person... I'm just saying something, or compared to all the people who use the app. I think that would motivate even more. [Woman, 64 years]*

*I liked that graph, the fact that you saw if it worked out well or not. I experienced that I tried to raise the bar a bit, by formulating actions that were a bit more challenging. Yes, I liked that. [Man, 29 years]*

*Looking back was sometimes confronting. On the other hand, it is satisfying when you have reached your goal. In that sense, that module is certainly an added value. [Woman, 28 years]*

### Overall recommendations of the users

During the interview, users were also asked if they had any recommendations to increase acceptability and feasibility of the ‘MyDayPlan’ app. Some of the most frequently cited recommendations are the following: implementation of a pedometer or activity tracker to have the ability to constantly track steps/active minutes (7/20), implementing a social aspect to compare or share action plans, coping plans and results with family and/or friends (2/20). Furthermore, implementing some game elements such as leader board, rewards, … was put forward by many users to make the app more attractive and reduce attrition (8/20). Several users also indicated that they would benefit from additional push notifications during the day to remind them of their action and coping plan (4/20). Finally, some users indicated that they would appreciate more input coming from the app (3/20); they wanted the ‘MyDayPlan’ app to operate more as a kind of personal trainer who tells you what to do.

## Discussion

This study evaluated the feasibility and acceptability of the ‘MyDayPlan’ application. High attrition is common in technological and web-based health intervention research, reflecting challenges in maintaining use of such technologies [43, 44]. Therefore, it is important to evaluate the potential end-users’ thoughts and experiences during development of the intervention [45]. Users’ thoughts and impressions were elicited through semi-structured interviews after having used the app in their everyday lives for 2 weeks. The results revealed that there are some important lessons to be learned.

Users of ‘MyDayPlan’ appreciated the time efficiency and ease of use of the app. Over the past years, we have substantially and iteratively invested in making our eHealth interventions more efficient and more easy to use, also taking into account suggestions of end users [25, 42]. In fact, in previous semi-structured interviews, some users suggested that a mobile app may further increase time efficiency and ease of use. The current results corroborate this statement. The ease of use was reported as an important factor contributing to acceptance of apps for health-care. It is likely that acceptance will decrease attrition [44, 46–48]. This is in line with results of a systematic review showing that the average attrition rate reported in app interventions is lower compared to average attrition rates in web-based interventions [49].

This study identified some challenges that will need to be further addressed. First, users should have the ability to navigate more easily between screens to correct and adjust. Second, many users indicated that minimizing typing texts into the app would be a substantial improvement. This may be addressed by providing more examples to select from. It is however unlikely that more examples will work in all situations. Third, next to hindering two individuals to participate in this study, technical failure of the app led to frustration of those experiencing it during the study. Therefore, pretesting the app in a variety of smartphones is a prerequisite for any mHealth intervention. Fourth, most users indicated appreciating the simple layout of ‘MyDayPlan’. This is also in line with previous research that has reported ease of use of a smartphone app as a key determinant of acceptance of the app [48]. However, a minority (20%) of the users indicated the simple layout as a negative point making the app not very attractive to use. These findings indicate large inter-individual differences in lay-out perception, revealing a challenge to meet the various expectations. Developing an up-to-date, attractive lay-out without sacrificing ease of use could be essential.

Of particular interest to this study were the experiences and opinions of users about the self-regulation components. Overall, the action planning component was considered feasible and acceptable. Most users stated that thinking about specific actions was helpful in selecting possibilities to be more physically active. We had expected that making action and coping plans for the upcoming day, would be easy. During a one-day cycle, participants may take the specific context of that day (working day, traveling, rainy weather,…) better into account. Feedback of users on the action planning component also indicated that some users wrongly interpreted the assignment. They thought they had to come up with a new, extra, original, creative but achievable action each day. The objective, however, was that users reflect upon possible PA actions that they can do within their daily routine. It was certainly not required to come up with (a) different action(s) each day. Providing clear and specific instructions for and expectations about the action planning is key for future users of ‘MyDayPlan’. Also the ability to copy and paste earlier action plans could be an added value.

Although users were not instructed to formulate different actions each day, some indicated that it was easy to come up with specific actions in the beginning, but that it became more difficult to formulate original, achievable but challenging actions. Therefore, in line with recommendations of some users, more examples could be provided in the action planning module. Users could have the option to select pre-formulated actions or formulate actions themselves. By doing so, users may come up with high-quality actions. Furthermore, earlier research showed that constantly varying and tailored pre-formulated actions would be more beneficial and effective than fixed, ‘one-size-fits-all’ pre-formulated actions [50].

In contrast to the action planning component, the coping planning component (barrier identification/problem solving) was perceived as less favourable. Most users experienced the making of coping planning as a difficult and unhelpful task. Many users struggled with identifying barriers and finding solutions. Some users indicated that thinking about possible action plans already resulted in the identification of possible barriers and solutions. Evidently, they experienced the coping planning as unnecessary. These findings are in contrast with earlier research, stating that coping planning is experienced as feasible and effective [51]. This could be explained by the fact that this is one of the first PA mHealth interventions implementing coping planning on a daily level. Therefore, the need for and effectiveness of the coping planning component within a daily micro cycle should be further investigated. Furthermore users should be guided more during the coping planning component by providing more information about how to formulate a high-quality coping plan and by providing the option to select a pre-formulated coping plan on top of the option to formulate coping plans themselves. To minimize the burden of the users, the coping planning could be limited to a general coping plan for the whole action plan instead of different coping plans for each specific action that was formulated/selected. Other possibilities are to require coping planning only when action plans of the day before were not executed.

The last self-regulation component, reviewing goal achievement, was highly appreciated and was perceived as very stimulating and motivating. However, users indicated that expanding this towards a continuous self-monitoring component, would help them to execute their set action plan by reminding them and confronting them with their behaviour throughout the day. This is in line with an earlier meta-analysis of Michie et al. evaluating effectiveness of several behaviour change techniques in interventions to increase PA in an adult population. This review has shown that self-monitoring is one of the most important behavioural change techniques to bridge the intention-behaviour gap [52]. In the current ‘MyDayPlan’ intervention, self-monitoring was barely implemented. It was limited to looking back on the action plan during the evening session and the ability to consult the graph that shows the extent to which the action plans have been carried out in the last 7 days. In line with general recommendations of the users, self-monitoring PA such as active minutes or MVPA with an activity tracker can increase the chance of effectively changing the PA behaviour of the users. This was confirmed in a review reporting evidence that self-monitoring through wearable activity trackers can increase PA levels in adult populations [53].

Most users indicated that the ‘MyDayPlan’ intervention was relevant for them. They were motivated by using the app and were made more aware of their level of PA and the need to be more physically active. However, most users indicated that they thought the app would not help them to effectively increase their level of PA on the long term. Indeed, ‘MyDayPlan’ is an intensive intervention, in which participants are required to make and execute plans during a relatively long period on consecutive days. Therefore, adaptations should be made to make daily use less intensive. This could go hand in hand with further increasing ease of use and time efficiency. The next step in this research is to make several adaptations to ‘MyDayPlan’ based on this study and to evaluate its effectiveness using an ABAB reversal design. Of note, ‘MyDayPlan’ is designed for research purposes and it is not the intention to make the application commercially available. However, this research could inform the development of future commercially available apps. Furthermore, an important future aim is to make this app available for the general public through governmental health-promoting initiatives by agencies such as Flemish Institute Healthy Living, LOGOs, health insurance agencies,… .

One of the strengths of this study is that only participants who did not meet the weekly PA recommendations of 150 min of weekly MVPA, were included. Also, we believe that our sample is representative for the potential target population of this intervention, making their opinions on the ‘MyDayPlan’ app very valuable. Second, the semi-structured interview was conducted after using the app in a free living context. This increases the ecological validity of the feasibility and acceptability results of this study.

Our study has also some limitations. First, we used a small, convenience sample with predominantly women (65%). This is in line with earlier research, indicating that women are more interested in health-related information and are proactive and engaged in seeking and gaining this information [54]. However, this limits generalizability of our findings, especially to the male population. Second, ‘MyDayPlan’ focusses on the post-intentional phase of the HAPA model, and, hence, is suited for users who have an intention to be more physically active. For those who have no intention to adopt an active lifestyle, the intervention may be less relevant, making their opinions and experiences less useful. Finally, accessibility and feasibility of ‘MyDayPlan’ was only evaluated based on qualitative data from the semi-structured interviews. Within this study, participants were constantly encouraged to fully use the app and complete all of the sessions. Furthermore they were contacted if they skipped a session to motivate them to engage in the following sessions. Consequently, quantitative data of this study on session completion and drop out would not correctly reflect adherence to the app, making it useless to evaluate feasibility. Future acceptability and feasibility studies however, should combine qualitative data with quantitative data on adherence (e.g. how many morning/evening sessions were attended?/ how many times was the app accessed?) in order to more comprehensively evaluate feasibility and acceptability.

## Conclusion

As ‘MyDayPlan’ is one of the first PA interventions implementing several self-regulation techniques using a one-day cycle, evaluating its feasibility and acceptability is essential before evaluating the effectiveness. This study revealed that ‘MyDayPlan’ is well-received and seems to be feasible and acceptable in a general adult population that does not meet the PA recommendations of 150 min of MVPA per week. However, some challenges remain, which will need to be addressed. Recommendations are the implementation of an activity tracker to self-monitor PA, increasing intuitiveness by minimizing text input of the users and provide more pre-programmed options and finally, provide more guidance for the coping planning component by providing more tailored examples. All these recommendations will be addressed and implemented in a next version of ‘MyDayPlan’.

## Supplementary information

**Additional file 1.** Interview guide.

**Additional file 2.** Coding Scheme.

**Additional file 3.** Screenshots of ‘MyDayPlan’.

## Data Availability

The datasets used and/or analysed during the current study are available from the corresponding author on reasonable request.

## Abbreviations

PA: Physical Activity
MVPA: Moderate to Vigorous Physical Activity
HAPA: Health Action Process Approach
IPAQ: International Physical Activity Questionnaire
IPAQ-LF: International Physical Activity Questionnaire Long-Form
