# Supplementary material for: Acceptability and feasibility of the mHealth intervention ‘MyDayPlan’ to increase physical activity in a general adult population
Source: BMC Public Health. 2020 Jun 29;20:1032. doi: 10.1186/s12889-020-09148-9 (PMC7325032; doi:10.1186/s12889-020-09148-9)
Supplement: Supplementary file 1 — Additional file 1. Interview guide. [file 12889_2020_9148_MOESM1_ESM.docx]

# Interview Guide

## Design of the app

### Perception of the app

- In general, how do you feel about the app?
- How did you experience using the app?
- To what extent, did you perceive the app as engaging?
- To what extent, did you think using the app was a fun thing to do?
- Could you easily keep your attention to the app?
- Which parts of the app did you like the most?
- Which parts of the app did you like the least?
  - Did you like creating an action plan?
  - Did you like searching for barriers and solutions?
  - Did you like reviewing your daily goal?

### User-friendliness

- In general, was the app easy or difficult to use?
- Which parts of the app did you experience as easy?
- Which parts of the app did you experience as difficult?
- Did you perceive creating your own action plan as easy/difficult?
- Did you perceive searching for barriers and solutions as easy/difficult?
- Did you perceive reviewing your goal as easy/difficult?
- How do you think about the user-friendliness of the app?
  - Could you easily find what you needed?
  - Where there moments that you were stuck?
  - Could you easily read all the text?

### Time efficiency

What do you think about the time you needed to complete the sessions?

- - In the morning
  - In the evening

### Lay-out

What do you think about the lay-out and the design of the app?

## Usefulness of the website

### Personal relevance

- Did you perceive the app as personal relevant?

### Awareness

- To what extent did the app make you more aware of your behaviour?

### Stimulating value of the app

- To what extent did the app help you to be more physically active?
- Which parts of the app helped you the most?
- Which parts of the app helped you the least?
  - Was it helpful to create an action plan?
  - Was it helpful to search for barriers and solutions?
  - Wat is helpful to review your goal?

### Informing value of the app

- To what extent did you learn new things by using the app?
- To what extent did you learn new things regarding behaviour change?
- To what extent did you learn new things regarding being more physically active?

## Recommendations

- Do you suggest any adaptations to the app?
- Are there things you would recommend us to change regarding the app?
