# Supplementary material for: Acceptability and feasibility of the mHealth intervention ‘MyDayPlan’ to increase physical activity in a general adult population
Source: BMC Public Health. 2020 Jun 29;20:1032. doi: 10.1186/s12889-020-09148-9 (PMC7325032; doi:10.1186/s12889-020-09148-9)
Supplement: Supplementary file 2 — Additional file 2. Coding Scheme. [file 12889_2020_9148_MOESM2_ESM.docx]

# Coding scheme

1. Design of the app
   1. General perception of the app
   2. Fun element
   3. User friendliness
   4. Time-efficiency
   5. Lay-Out
2. Content of the app
   1. Personal relevance
      - 1. Action planning
        2. Coping planning
        3. Review
   2. Stimulating value
      1. Awareness
         1. Action planning
         2. Coping planning
         3. Review
      2. Help towards behaviour change
         1. Action planning
         2. Coping planning
         3. Review
3. Recommendations
   1. Monitoring (activity tracker)
   2. Game elements (leader board)
   3. Social module
   4. Tailored pre-programmed action/coping plans
