# Supplementary material for: Acceptability and feasibility of the mHealth intervention ‘MyDayPlan’ to increase physical activity in a general adult population
Source: BMC Public Health. 2020 Jun 29;20:1032. doi: 10.1186/s12889-020-09148-9 (PMC7325032; doi:10.1186/s12889-020-09148-9)
Supplement: Supplementary file 3 — Additional file 3. Screenshots of ‘MyDayPlan’. [file 12889_2020_9148_MOESM3_ESM.docx]

# Screenshots of ‘MyDayPlan’


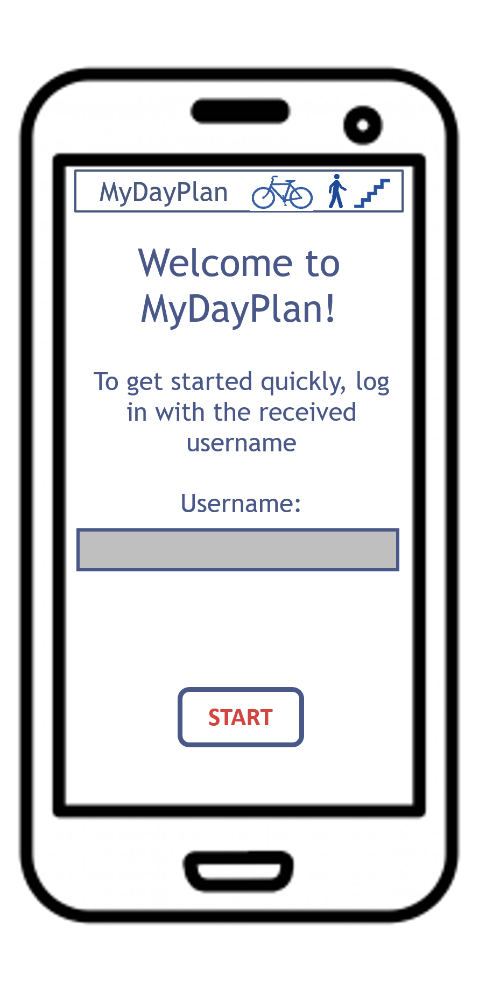

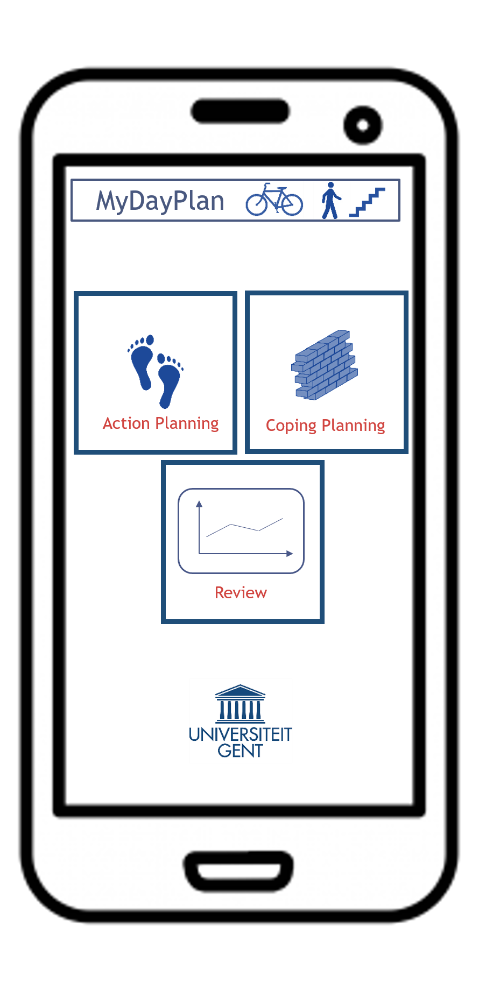


## Action Planning (Morning)


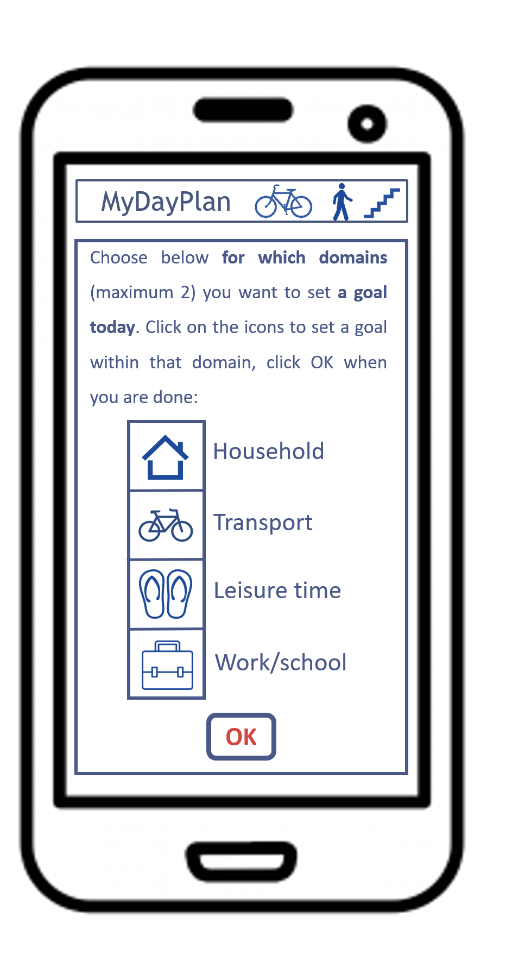

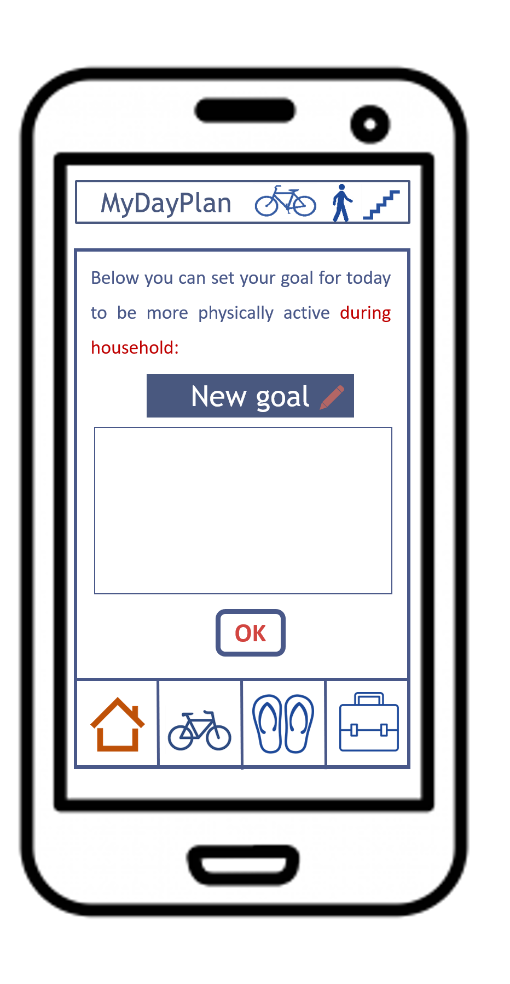

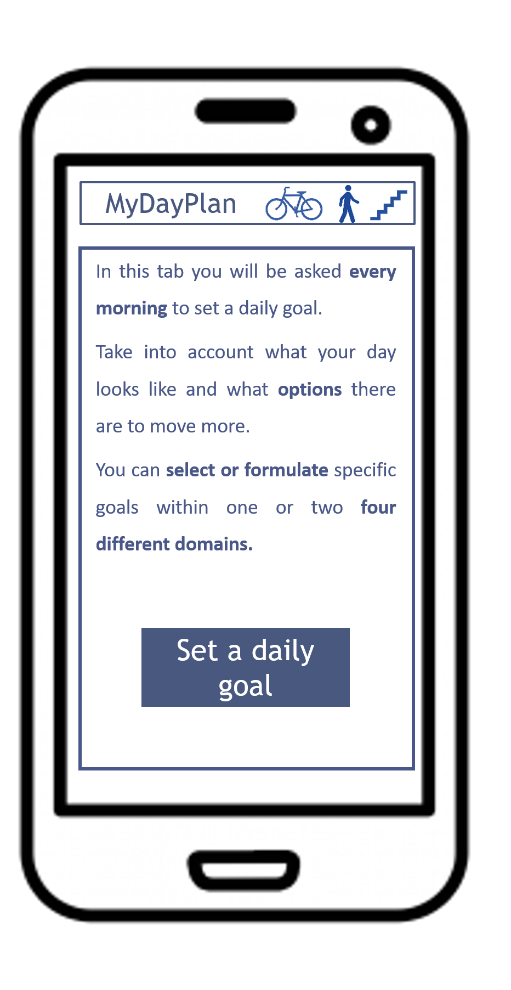


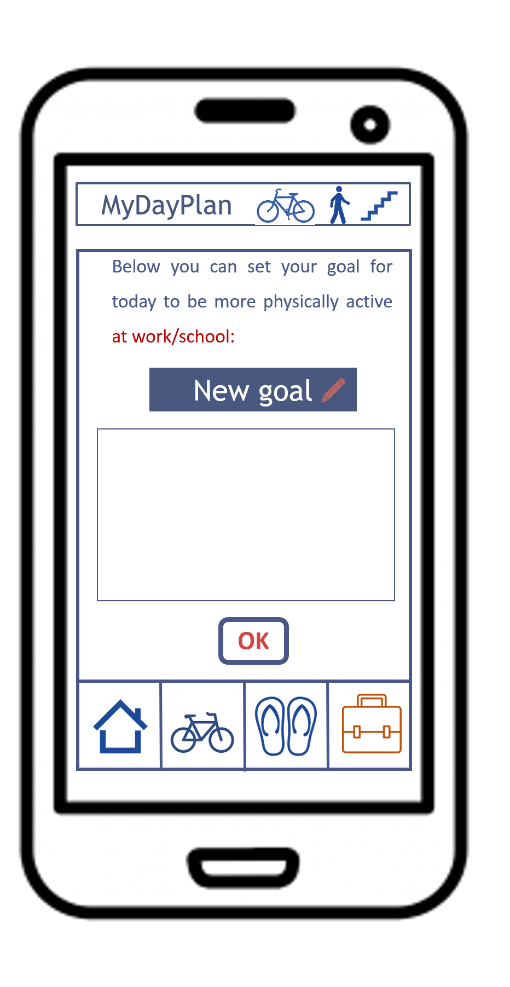

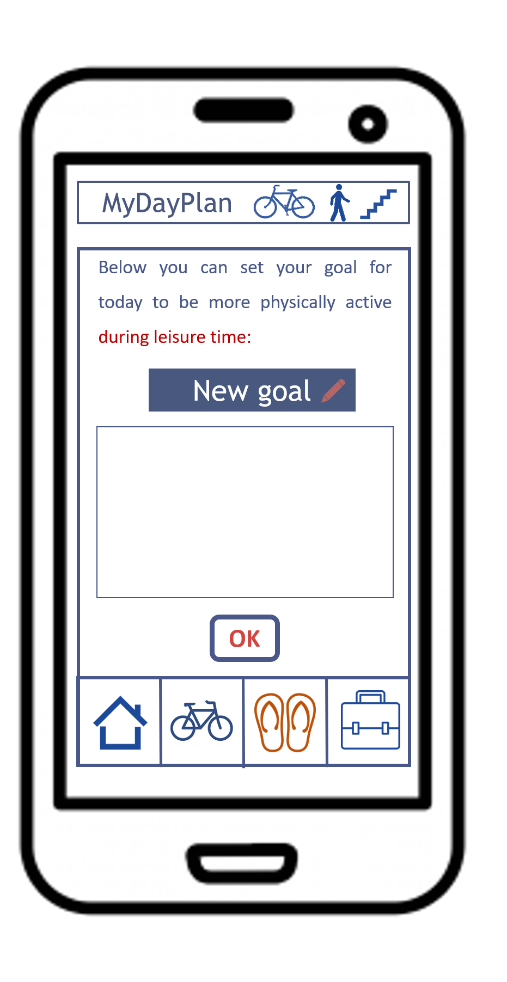

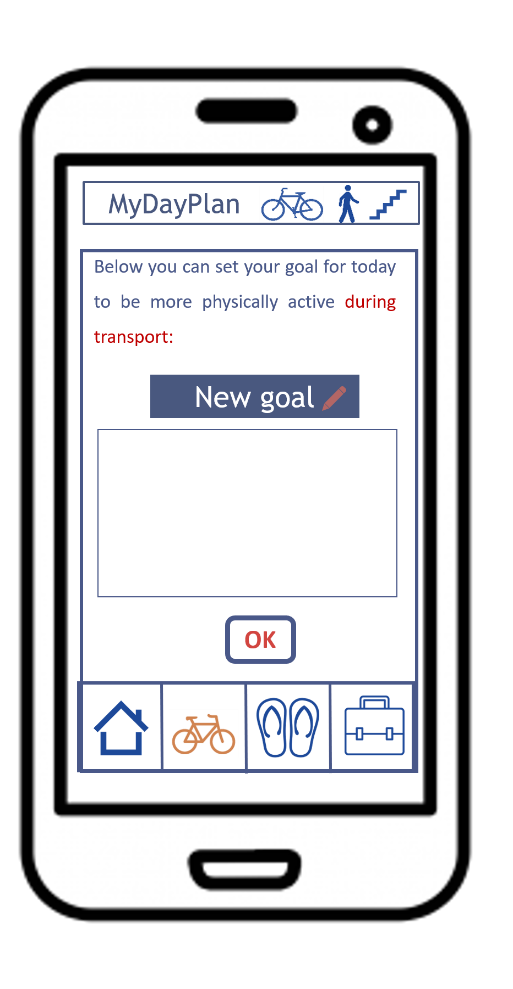


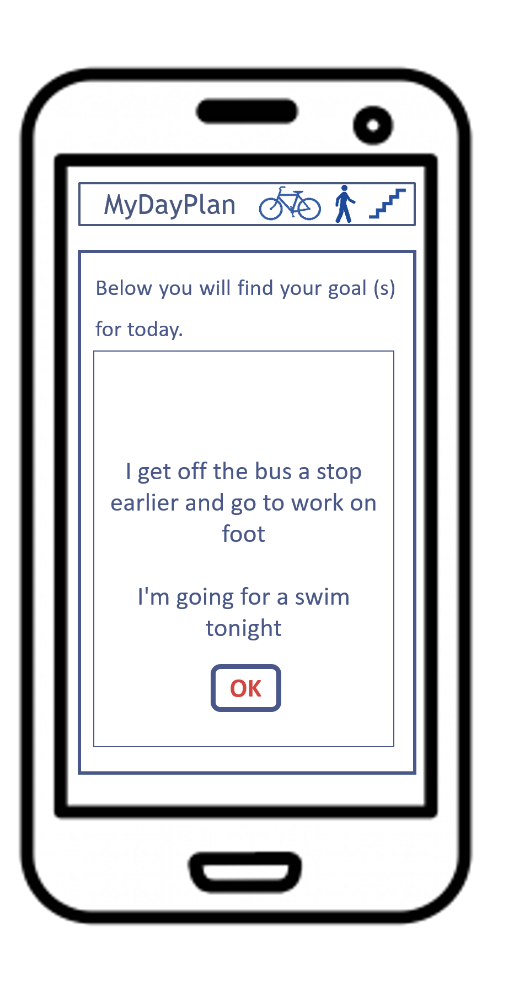


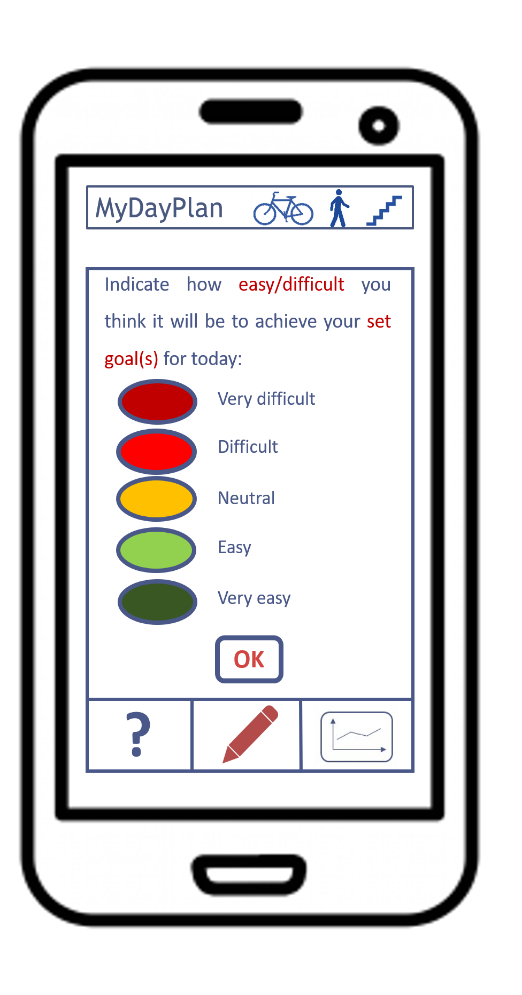


## Coping planning (Morning)

##
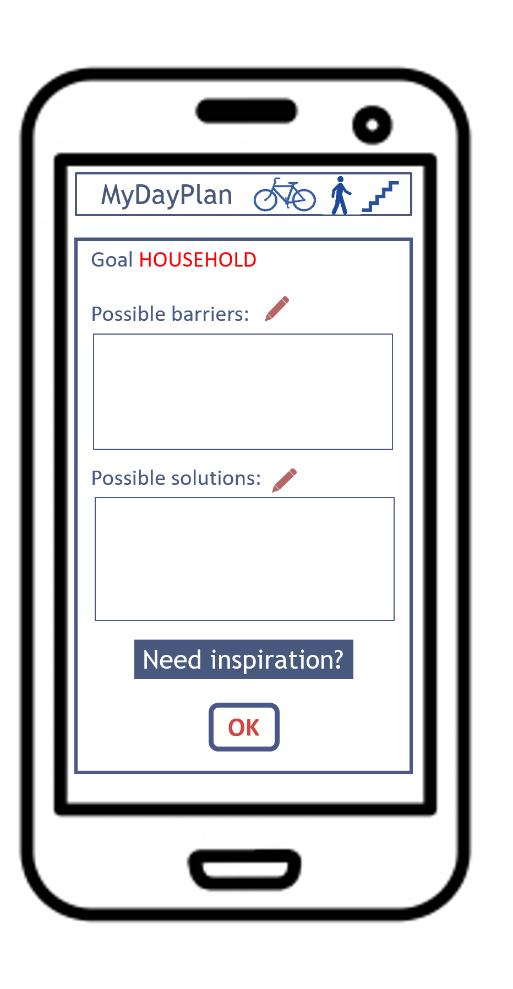

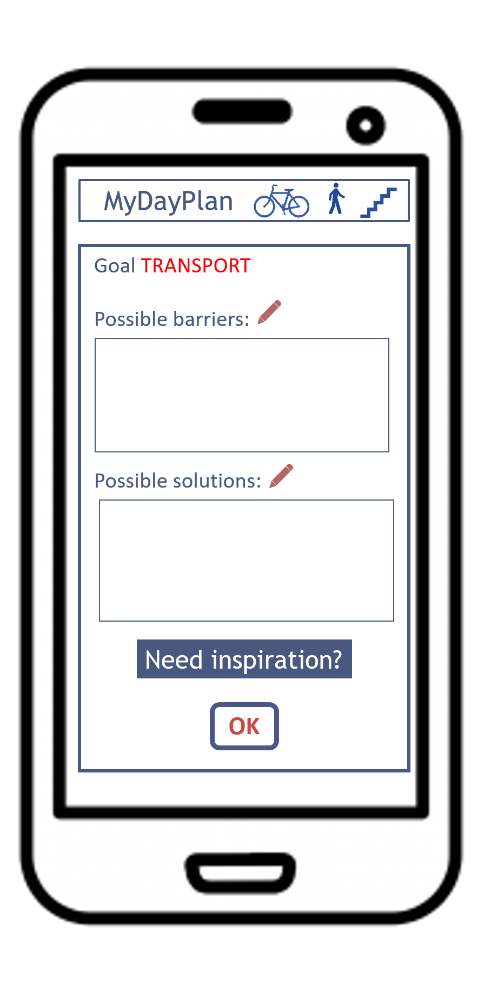


##
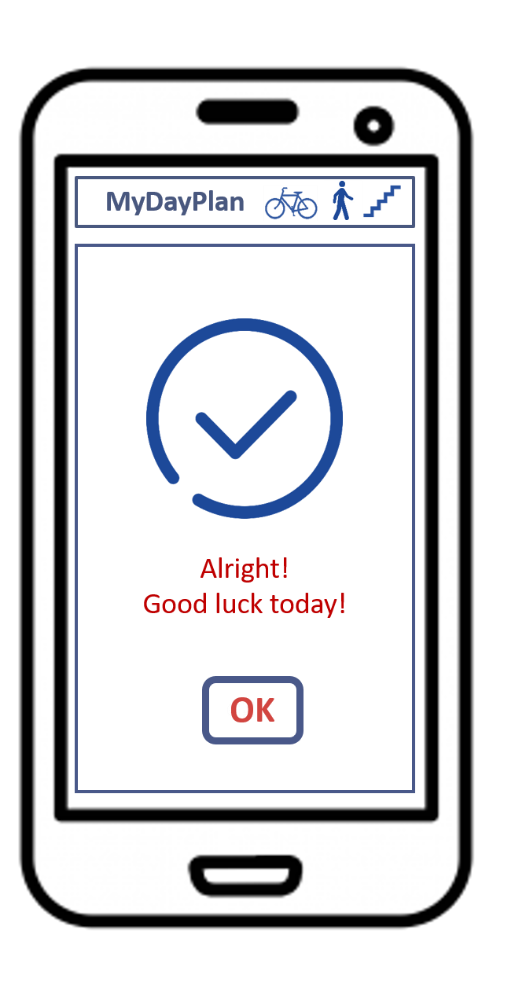

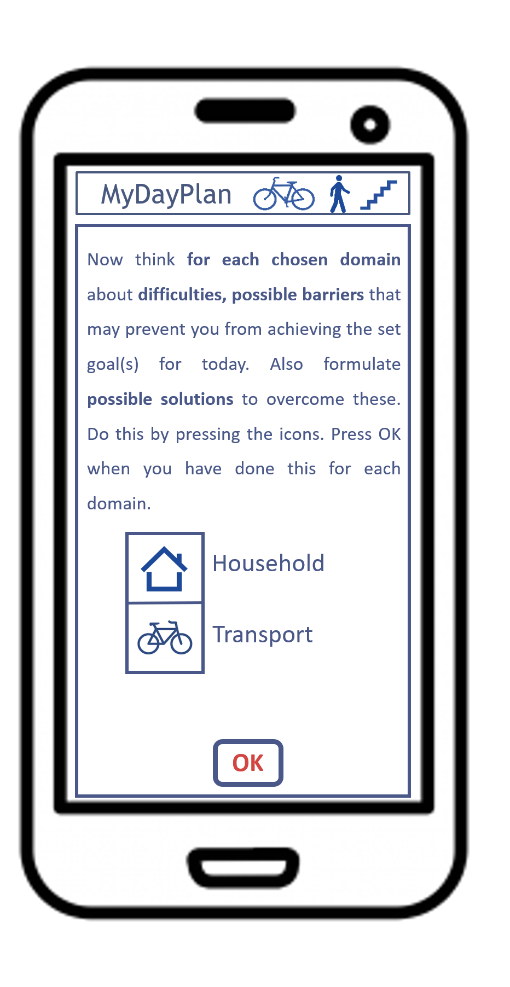


##
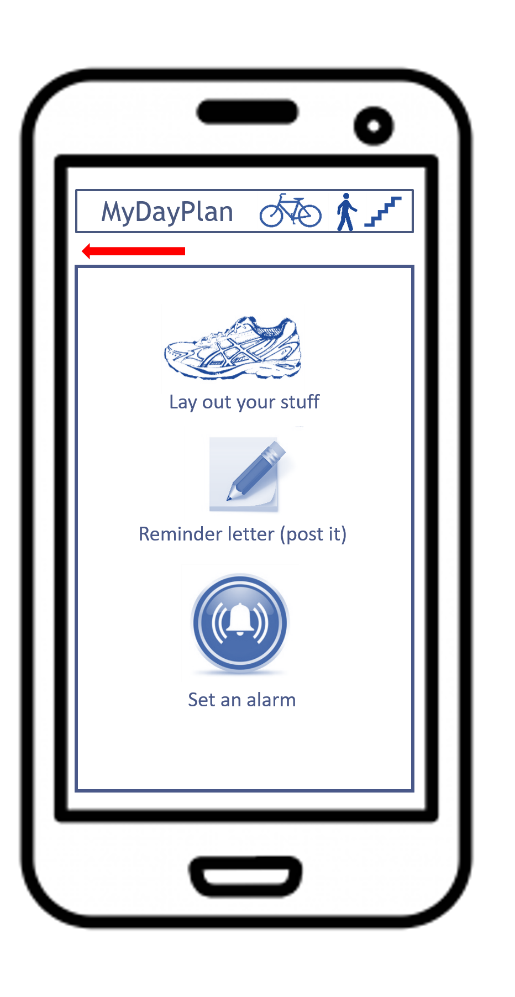

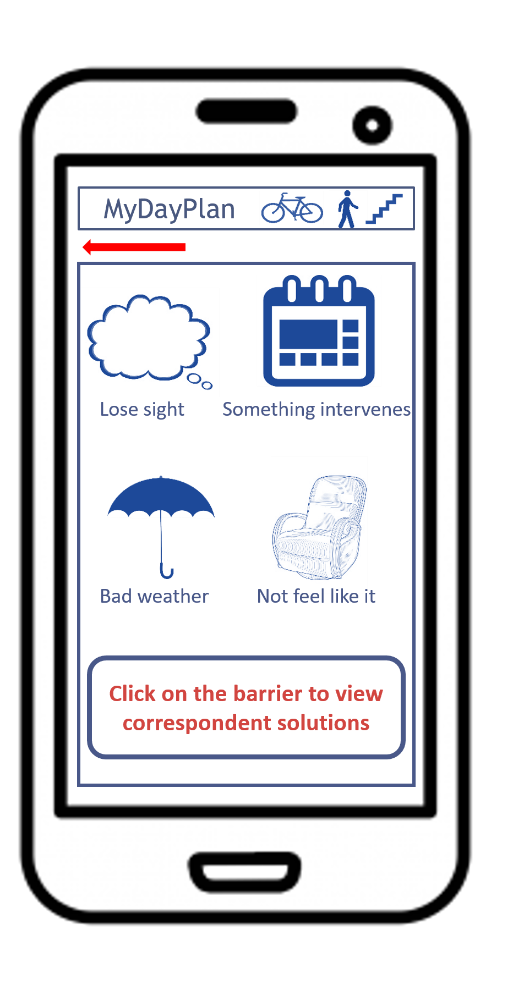


## Review (Evening)

##
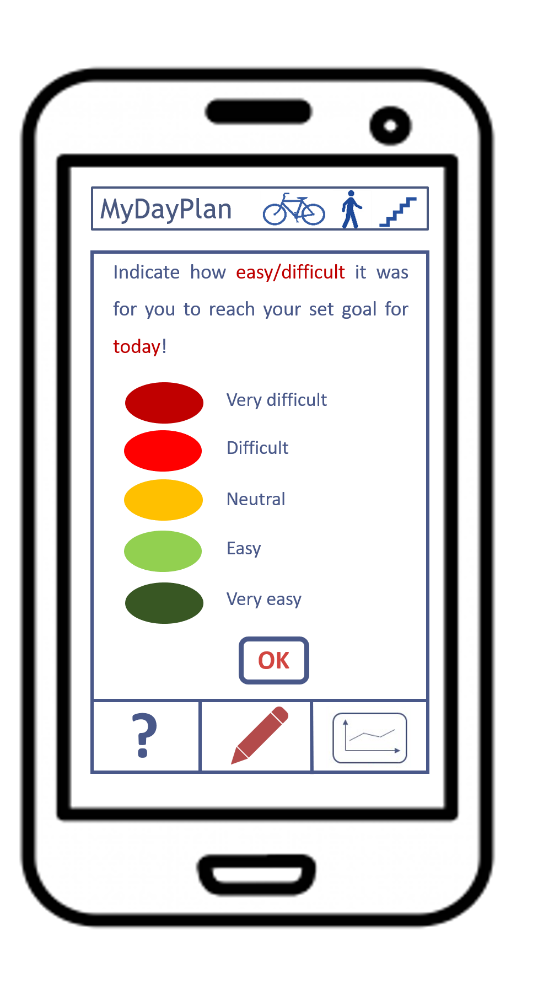

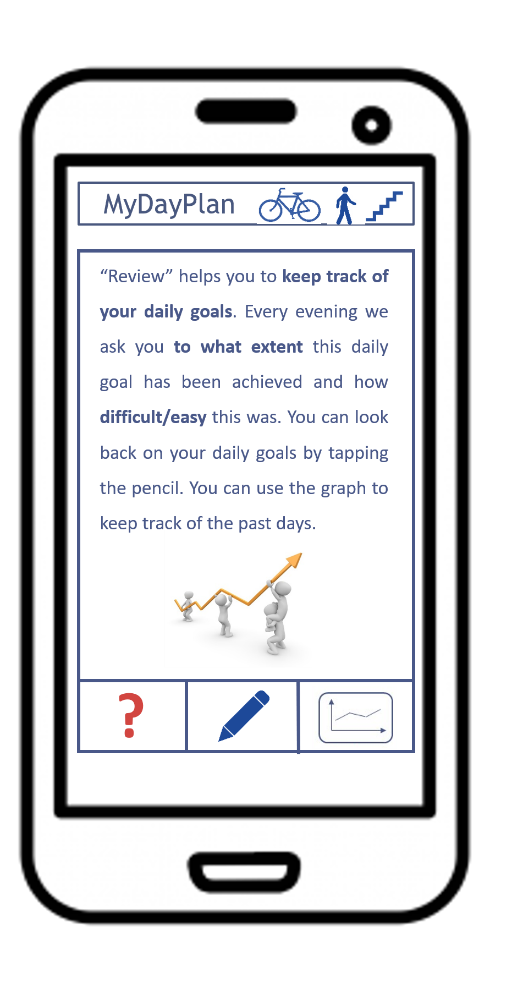


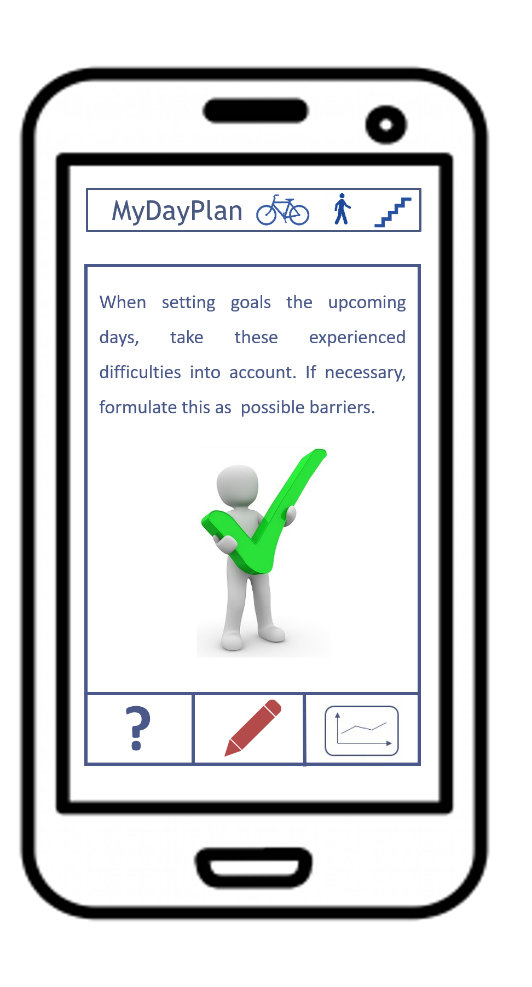

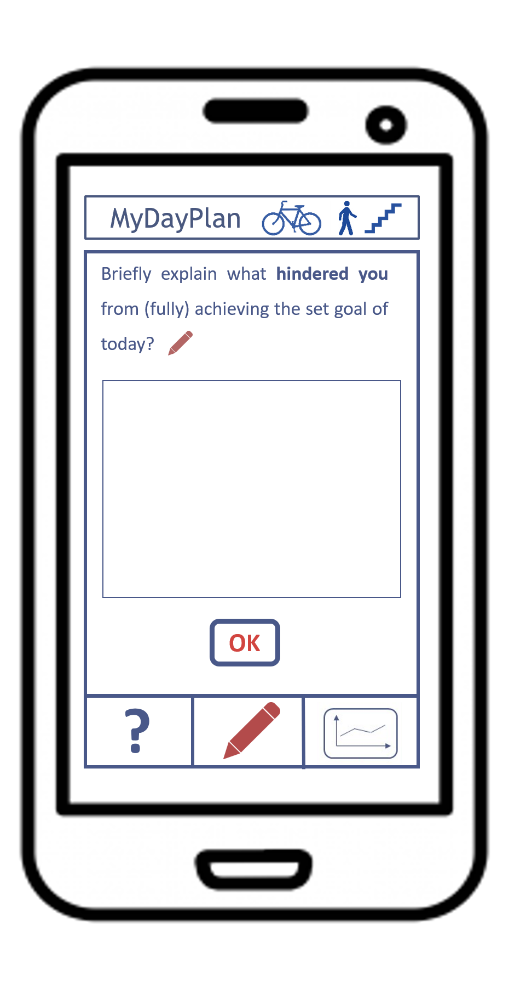

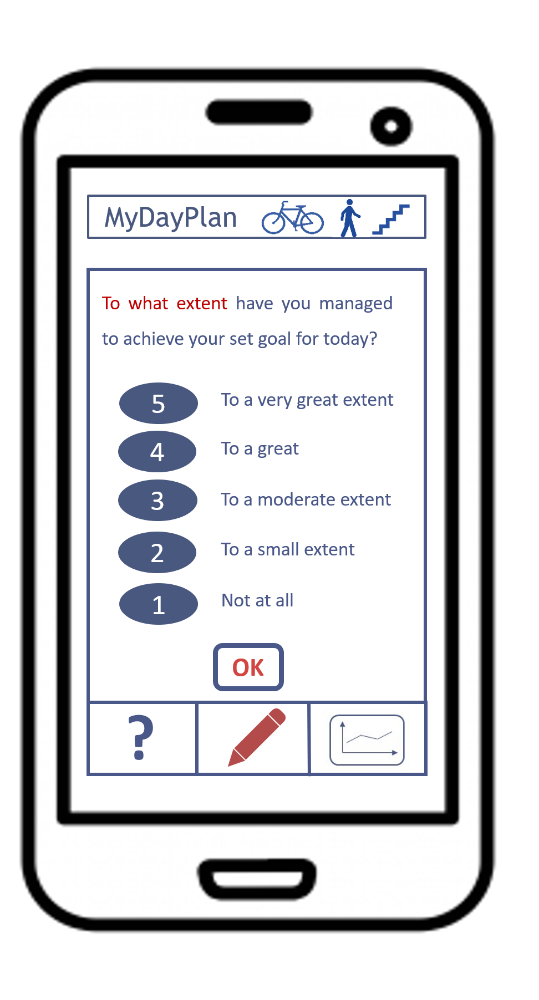


If 1,2 or 3 is chosen


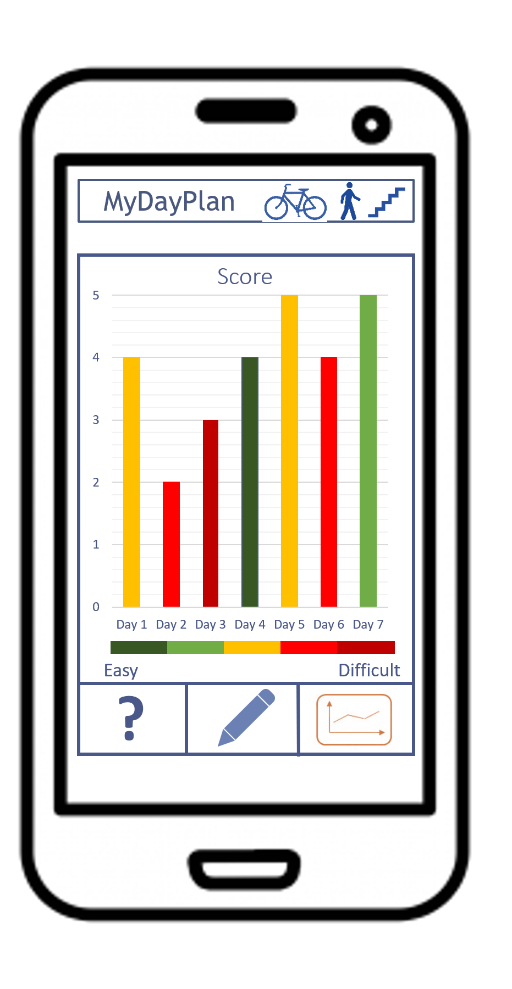

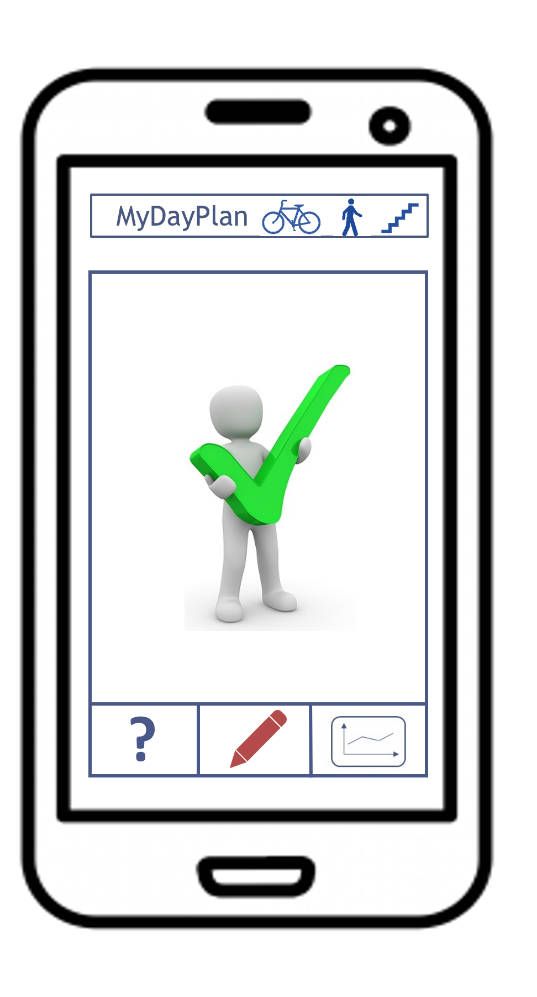

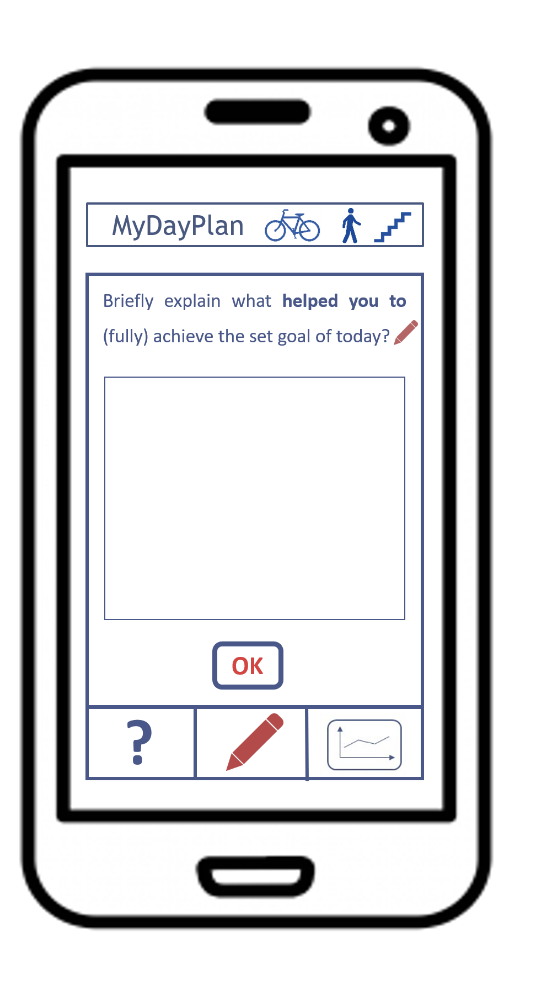


If 4 or 5 is chosen
